# Effect of diotic versus dichotic presentation on the pitch perception of tone complexes at medium and very high frequencies

**DOI:** 10.1038/s41598-023-40122-8

**Published:** 2023-08-15

**Authors:** Hedwig E. Gockel, Robert P. Carlyon

**Affiliations:** MRC Cognition and Brain Sciences Unit, Cambridge Hearing Group, University of Cambridge, 15 Chaucer Road, Cambridge, CB2 7EF UK

**Keywords:** Neuroscience, Physiology, Psychology

## Abstract

Difference limens for fundamental frequency (F0), F0DLs, are usually small for complex tones containing low harmonics that are resolved in the auditory periphery, but worsen when the rank of the lowest harmonic increases above about 6–8 and harmonics become less resolved. The traditional explanation for this, in terms of resolvability, has been challenged and an alternative explanation in terms of harmonic rank was suggested. Here, to disentangle the effects of resolvability and harmonic rank the complex tones were presented either diotically (all harmonics to both ears) or dichotically (even and odd harmonics to opposite ears); the latter increases resolvability but does not affect harmonic rank. F0DLs were measured for 14 listeners for complex tones containing harmonics 6–10 with F0s of 280 and 1400 Hz, presented diotically or dichotically. For the low F0, F0DLs were significantly lower for the dichotic than for the diotic condition. This is consistent with a benefit of increased resolvability of harmonics for F0 discrimination and extends previous results to harmonics as low as the sixth. In contrast, for the high F0, F0DLs were similar for the two presentation modes, adding to evidence for differences in pitch perception between tones with low-to-medium and very-high frequency content.

## Introduction

Research on human pitch perception has shown that difference limens for the discrimination of fundamental frequency (F0), F0DLs, are usually small for complex tones containing resolved harmonics, and start to increase (worsen) when the rank of the lowest harmonic increases above about 6–8. F0DLs tend to reach a high plateau when only harmonics with very high ranks (above about 14–16) are present^1–4^.

One explanation for this pattern of results involves the resolvability of the components. The frequencies of the components of a harmonic complex tone are integer multiples of the F0 and thus are linearly spaced in frequency. In the cochlea, these components are separated to some extent, i.e. each harmonic gives rise to a maximum of vibration (excitation) at a specific place on the basilar membrane (BM). Each place on the BM behaves like a nonlinear bandpass filter, with tonotopical arrangement of the filters’ center frequencies (CFs) on the BM. However, the bandwidths of these filters increase with increasing center frequency^5^, and thus with increase in harmonic rank the components become less resolved on the BM and the vibration pattern at the output of the filter becomes more complex. Auditory neurons tend to fire at a particular phase of the waveform, and so the change in vibration pattern is reflected in a change in the temporal firing pattern of auditory nerve fibres, as well as in the distribution of firing rates across fibers with different CFs.

Although the mechanisms underlying pitch perception remain debated^6^, it is generally accepted that pitch is usually extracted by a central mechanism that combines place-of-excitation information (i.e. the distribution of firing rates across auditory nerve fibres tuned to different frequencies) and/or temporal (phase locking) information about the frequencies of the resolved harmonics^7^. When the rank of the lowest harmonic is increased above 6–8, the harmonics become less resolved, and pitch may then be extracted from the temporal fine structure (TFS) of the waveform evoked in the cochlea (when the rank of the lowest harmonic is in the range from about 6 to 16)^8,9^. For even higher harmonic ranks, components become completely unresolved and pitch may be extracted from the repetition rate of the envelope. The pitch of a complex tone containing only unresolved harmonics is less clear than that of a complex containing resolved harmonics, presumably because the broader envelope peaks of the vibration pattern provide for less precise temporal (phase locking) information than the information derived from (partially) resolved harmonics that produce narrower peaks in the pattern of excitation, and that can be based on either the place-of-excitation or temporal information. The present study concerns the increase in F0DL with increasing harmonic rank that occurs within the range of (partially) resolved harmonics, rather than the difference between the generally low F0DLs for resolved harmonics versus the much higher F0DLs associated with the “envelope pitch” of complexes that contain only very high-numbered harmonics.

A second explanation for the increase in F0DLs as the harmonic number of the lowest harmonic increases, suggested by Bernstein and Oxenham^10,11^, is that the key factor is harmonic rank, rather than resolvability. It may be that the pitch mechanism extracts information most effectively from places in the cochlea tuned to frequencies that are not more than 6–10 times the perceived F0^12,13^. Bernstein and Oxenham^11^ showed that a 3% increase of the F0 of the odd harmonics relative to that of the even harmonics, which led to perceptual segregation of the odd and even harmonics, significantly reduced the F0DLs for a complex tone containing only high unresolved harmonics, even though resolvability was little affected by this manipulation. The pitch of the complex was dominated by the even harmonics, whose F0 is double that of the complex as a whole, and Bernstein and Oxenham^11^ suggested that the resulting halving of the effective harmonic ranks for the even harmonics led to the reduction in F0DLs.

A possible problem with an explanation based entirely on harmonic ranks comes from another finding of Bernstein and Oxenham^11^ that F0DLs decreased when the odd and even harmonics were presented to opposite ears (referred to hereafter as dichotic presentation). Dichotic presentation increases resolvability because the harmonics in each ear are more widely spaced in frequency than for diotic presentation, but it does not affect harmonic rank at least when harmonic rank is expressed relative to the F0. Hence, this result suggests that resolvability does play some role. However, the important factor might be the harmonic rank expressed relative to the *perceived pitch*, which might not correspond to the F0 and which might change with dichotic presentation. To check on this, Bernstein and Oxenham^11^ obtained pitch matches to the dichotic complexes. The decrease in F0DLs for dichotic presentation was observed only when the complex tones contained no harmonic below the 9th^11^. The pitch matches showed that when the lowest audible harmonic had a rank of 9 or lower, the perceived pitch clearly corresponded to the F0, while when the lowest audible harmonic had a rank of 13 or higher, the pitch clearly corresponded to 2F0. Thus, for a complex tone whose lowest audible harmonic had a rank of 9, F0DLs were clearly lower in the dichotic condition than in the diotic condition, while the overall pitch and thus the effective harmonic ranks were unchanged. Taken together, the results of Bernstein and Oxenham’s experiments are consistent with both the perceived ranks of the harmonics and the resolvability of the harmonics affecting the accuracy of pitch perception.

Unfortunately, dichotic presentation of harmonic complexes may affect F0DLs in ways other than by changing resolvability or effective harmonic rank. For example, Bernstein and Oxenham^11^ argued that when the assignation of odd- and even-numbered harmonics to each ear is fixed across presentations within trials (the “fixed paradigm”), listeners may use the increased frequency spacing to track changes in the frequencies of individual harmonics. This cue, referred to hereafter as frequency tracking, may have been used in the study of Bernstein and Oxenham^11^, which employed the fixed paradigm, and this might explain the decrease in F0DLs for dichotic relative to diotic presentation, without reflecting a change in the processing of the pitch of the complex as a whole. Additionally, even when listeners judge the pitch of a dichotic complex to correspond to the F0 of the combined stimulus to the two ears, consistent with them integrating harmonics across ears, they might perform a discrimination task using the pitch derived from the harmonics presented to just one ear, if that yields better performance. That is, the dominant pitch may correspond to the whole dichotic stimulus but the listener might be able to access the information provided by each ear separately.

Recent studies have compared F0DLs for diotic and dichotic presentation using complex tones that contained only harmonics with very high frequencies (≥ 8.4 kHz) with low to intermediate harmonic numbers (harmonics 6–10 of a 1400-Hz F0)^14,15^. At these very high F0s and for a stimulus duration of 210 ms, frequency tracking would be unlikely to reduce F0DLs, because frequency difference limens (FDLs) for individual harmonics in this very-high-frequency region are substantially higher than the F0DL for the whole complex^14,16^. Hence these very-high-frequency stimuli provide an opportunity to evaluate the effects of dichotic presentation without the potentially confounding effects of frequency tracking. The perceived pitch for these high-frequency complex tones did not double for dichotic presentation, but corresponded to the F0^17^. The studies of Lau et al.^14^ and Gockel and Carlyon^15^ also included a condition with an F0 of 280 Hz (again with harmonics 6–10). Both studies observed a trend for F0DLs to be lower for dichotic than for diotic presentation for both F0s. However, the effects were not significant, possibly because of substantial individual variability and the relatively small number of listeners, which was eight for Lau et al.^14^, and five for Gockel and Carlyon^15^.

The objective of the present study was to get a more definitive answer to the question of whether dichotic presentation leads to smaller F0DLs for tones containing harmonics with intermediate ranks, for which the pitch would not double with dichotic presentation, both in a low-to-intermediate frequency region and in a very-high frequency region. For this purpose, F0DLs were determined for a larger group of listeners using 210-ms complex tones containing harmonics 6–10 with an F0 of 280 Hz and an F0 of 1400 Hz.

## Results

**Figure 1:**
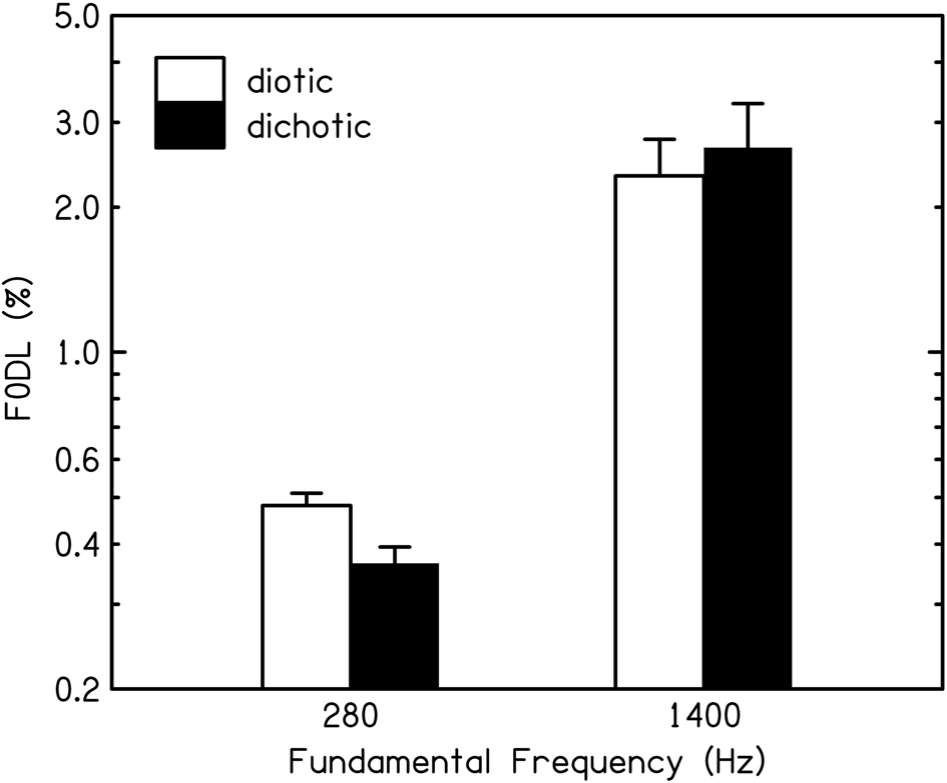
Geometric mean difference limens for fundamental frequency (F0DLs, and standard errors of the mean) of complex tones containing harmonics 6–10 for 14 listeners, for each fundamental frequency (F0) and each presentation mode. Diotic: all harmonics were presented to both ears; Dichotic: odd harmonics were presented to the left and even harmonics were presented to the right ear.

Figure 1 shows the geometric mean F0DLs. Error bars show ± one standard error of the mean (SEM). As expected, F0DLs were higher for the 1400-Hz than for the 280-Hz F0. More importantly, F0DLs were lower for the dichotic than for the diotic presentation mode for the 280-Hz F0 but they were similar for these two presentation modes for the 1400-Hz F0. Note that F0DLs are assessed on a logarithmic rather than a linear scale, because perceptual sensitivity to a change in a given (mean) F0 is proportional to the difference in F0 (in %), i.e. sensitivity increases by a factor of two if the difference in F0 (in %) increases by a factor of two. This relationship between sensitivity to small changes in F0 and the amount of change in F0 holds for both low- to medium and the very high frequencies used here^16^.

To assess the significance of the results, a univariate general linear model (an analysis of variance, ANOVA) was employed. Two fixed within-subjects factors (F0 and presentation mode), and one random factor (subject) were specified. The dependent variable was the logarithm of the F0DLs, which were approximately normally distributed. There was a highly significant main effect of F0 [*F*(1,13) = 89.95, *p* < 0.001, partial eta-squared (*η*_*p*_^2^) = 0.87] as expected. Neither the main effect of presentation mode [*F*(1,13) = 1.27, *p* = 0.280, *η*_*p*_^2^ = 0.09] nor that of subject [*F*(13,13.78) = 1.17, *p* = 0.387, *η*_*p*_^2^  = 0.53] were significant. Importantly, a highly significant interaction between F0 and presentation mode was observed, showing that the effect of dichotic presentation differed between the two F0s [*F*(1,13) = 16.40, *p* = 0.001, *η*_*p*_^2^ = 0.56]. In addition, subject interacted significantly with F0 [*F*(13,13) = 13.7, *p* < 0.001, *η*_*p*_^2^ = 0.93], showing that listeners’ relative performance differed between the two F0s, but there was no significant interaction between subject and presentation mode [*F*(13,13) = 1.53, *p* = 0.227, *η*_*p*_^2^ = 0.60].

The interest in this study was the effect of presentation mode at the two F0s, and therefore, and to further assess the nature of the significant interaction between F0 and presentation mode, a dependent-samples *t*-test (appropriate for matched samples as here) was calculated, separately for each F0, to compare the mean F0DLs in the diotic and the dichotic conditions. For the 280-Hz F0, F0DLs were significantly larger for the diotic than for the dichotic condition [*t*(13) = 3.92, *p* = 0.002, 2-tailed, Cohen’s *d* = 1.05], while for the 1400-Hz F0 there was no significant difference between the two modes of presentation [*t*(13) = − 1.5, *p* = 0.157, 2-tailed, Cohen’s *d* = − 0.4].

## Discussion

For the low frequency region, the present results confirm the finding of Bernstein and Oxenham^11^ that F0DLs decrease with dichotic presentation. The present results extend this finding to complex tones whose lowest harmonic was as low as the 6th; in the data of Bernstein and Oxenham^11^ a decrease in F0DLs for dichotic presentation was not observed when the lowest harmonic present was below the 9th. The decrease in F0DLs observed here was smaller than that observed previously for complex tones where the lowest harmonic rank was higher^10,11^. This can be expected, given that harmonics 6–10 are (partially) resolved and therefore here F0DLs in the diotic condition were already relatively small. However, harmonic resolvability falls on a continuum, and even though the sixth harmonic is often regarded as resolved, its internal representation would be affected by the presence of adjacent harmonics^18^.

For the high frequency region, F0DLs were similar for diotic and dichotic presentation. Hence in this condition, where the frequency tracking cue would not be of benefit, no dichotic advantage was observed. The absence of a dichotic advantage shows that, in this condition at least, neither increased resolvability nor a decreased effective harmonic rank in the ear receiving the even harmonics led to reduced F0DLs.

In the authors’ opinion, there are two classes of explanation for the finding of a dichotic advantage for the low-frequency but not the high-frequency condition. One of these is that there is only ever a dichotic advantage when listeners use frequency tracking, which, as argued above, would have reduced F0DLs only for the low region. This is consistent with the finding of Bernstein and Oxenham^10^ of no dichotic advantage when the rank of the lowest harmonic and the ear of presentation of the even and odd harmonics were randomized across intervals within a trial; this would make frequency tracking very difficult. However, the lack of a dichotic advantage in this randomized paradigm could be a consequence of the timbre differences between the tones to be compared; Moore and Glasberg^19^ showed that timbre differences between the tones that had to be compared negatively affected F0DLs. Therefore it is possible that the distracting within-ear changes in timbre in the randomized paradigm used by Bernstein and Oxenham^10^ offset an otherwise positive effect of increased resolvability in the dichotic condition.

The second class of explanation is that the factors influencing pitch perception and F0 discrimination differ qualitatively between the low and very high frequency regions. For example, Gockel et al.^16^ have argued that the weak or absent phase locking to the individual components of the high-frequency complex forced listeners to rely on excitation-pattern cues. For the frequency discrimination of an isolated pure tone (a single harmonic), the peak in the excitation pattern produced by that tone might be confused with random peaks in the excitation pattern produced by the background threshold equalizing noise (TEN; see “Methods” section). They argued that for a harmonic complex this confusion would be reduced because of the regular pattern of excitation peaks, leading to a larger decrease in the F0DL than predicted from the FDLs for the individual harmonics, based on an optimum-combination model. It is possible that these confusion effects limited discriminability even for the high-region complex tones used here. Dichotic presentation might lead to a better representation of peaks in the excitation pattern corresponding to the individual harmonics, but there would be fewer peaks within each ear to help resolve confusion, so the net effect might be no benefit. If so, this could account for the absence of a dichotic advantage for the high frequency region. Gockel et al.^16^ argued that these excitation-pattern confusion effects were less likely to limit performance at low frequencies, where phase locking is stronger, and so the limitations on F0 discrimination performance may well have differed between the two frequency regions. The significant interaction between subject and F0 that was observed here, is therefore also consistent with the explanation that different factors limit F0 discrimination in the low and very high frequency regions.

In summary, F0DLs were measured for complex tones containing harmonics 6–10 with F0s of 280 and 1400 Hz for diotic and dichotic presentation. For the low F0, F0DLs for the dichotic condition were significantly lower than for the diotic condition, thus extending previous findings down to even lower harmonic ranks. The reduction in F0DLs for dichotic relative to diotic presentation was small in comparison to the reduction observed previously for stimuli with higher lowest-harmonic rank, where dichotic presentation or perceptual segregation of the odd and even harmonics usually doubled the perceived pitch and thus halved the effective harmonic ranks.

In contrast, for the high F0, F0DLs were similar for the two modes of presentation. This finding may be due either to listeners being able to use frequency tracking in the low region only, and/or to differences in the factors limiting F0 discrimination in noise in the two regions. This study provides further support for a qualitative difference in how listeners process information when comparing the pitches of complex tones for stimuli with low- to medium frequency components and stimuli with only very high frequency components.

## Methods

### Participants

Fourteen young normal-hearing musically trained listeners (9 females) between 16 and 28 years of age (mean age of 21 years) participated. None of them was a professional musician. The average number of years of musical training was 13.9 (ranging from 5 to 21 years), with a standard deviation of 4.4 years. Most of them played a string instrument or sang in a choir. There was no fixed lower threshold for musical training at the time of subject recruitment, but subjects with musical training were sought in order to rule out conditions like amusia^20^ and to minimize learning effects during the course of the experiment^21^.

All listeners had to pass a three-stage screening, as in Lau et al.^14^ and as described in detail in Gockel and Carlyon^17^, to ensure audibility of the high-frequency tones and basic frequency discrimination ability. Briefly, the requirements for passing the screening were: (1) Pure-tone audiometric thresholds in quiet at octave frequencies from 0.25 to 8 kHz and at 6 kHz had to be ≤ 15 dB hearing level (HL). (2) Thresholds for detecting 210-ms sinusoidal tones at 10, 12, 14 and 16 kHz in a continuous threshold equalizing noise, TEN^22^, had to be ≤ 45 dB SPL up to 14 kHz, and ≤ 50 dB SPL at 16 kHz. The TEN was the same as used in the main experiment (see below). (3) F0DLs for diotically presented 210-ms complex tones containing harmonics 6–10 with an F0 of 280 or 1400 Hz (the same tones as used in the main experiment, except for the absence of level randomization; see below) and FDLs for the individual components of the complex tones presented in isolation had to be < 6% and < 20% in the low and high frequency regions, respectively. The adaptive procedure tracked 79% correct responses, and tones were presented in quiet.

Initially 36 musically trained listeners between 16 and 28 years old were tested, 14 of whom passed all screening stages. This study was approved by the Cambridge Psychology Research Ethics Committee and carried out in accordance with the Declaration of Helsinki and the UK regulations governing biomedical research. All experiments were performed in accordance with relevant guidelines and regulations. Written informed consent was obtained from all participants, and they were paid for taking part.

### Stimuli

F0DLs were measured for 210-ms (including 10-ms onset and offset hanning-shaped ramps) complex tones containing harmonics 6–10, with F0s of 1400 or 280 Hz. For each presentation, the starting phases of all components were randomized and individual component levels were roved over the range ± 3 dB (uniform distribution) about the mean component level, which was 55 dB SPL for harmonics 7–9 and 49 dB SPL for the edge components. In condition “diotic”, tones were presented diotically in a background of a continuous diotic TEN, extending from 0.02 to 22 kHz and with a level of 45 dB SPL/ERB_N_ at 1 kHz, where ERB_N_ stands for the average value of the equivalent rectangular bandwidth of the auditory filter for young normal-hearing listeners tested at low sound levels^23^. In condition “dichotic”, the odd harmonics were presented to the left and even harmonics were presented to the right ear, with an independent TEN at each ear. The TEN was used throughout the experiment in order to mask otherwise possibly audible distortion products that arise in the inner ear, and also to ensure that the individual harmonics had approximately equal sensation levels. These stimuli were the same as used by Lau et al.^14^ and by Gockel and Carlyon^15^, except that the former used gated rather than continuous TEN.

### Experimental procedure

A two-interval two-alternative forced-choice task with a 3-down 1-up rule was used. After each trial, listeners received feedback as to whether their response was correct or incorrect. The initial F0 difference was 20% and the F0s of the two tones were centered geometrically on the nominal F0. The initial step size was a factor of 2. This was decreased to a factor of 1.41 after two reversals, and to a factor of 1.2 after four reversals. Eight more reversals were collected and their geometric mean used as the threshold estimate, tracking the F0 difference needed for listeners to score 79% correct. At least five but usually six threshold estimates were obtained for each listener and condition, and the final threshold was the geometric mean of the threshold estimates for the last five adaptive tracks for each condition. The presentation order of the four conditions (two F0s: 280 and 1400 Hz; two presentation modes: diotic and dichotic) was counterbalanced across repetitions within and across listeners. Data collection took on average four sessions of 2 h each (including breaks) for each listener.

For statistical analysis, a repeated-measures analysis of variance (RM-ANOVA) was calculated using SPSS (Chicago, IL; Version 27) with two fixed within-subjects factors (F0 and mode of presentation) and subject as a random factor. Before statistical analysis, the F0DLs were log-transformed to make them more normally distributed. Shapiro–Wilk tests confirmed that the (transformed) data were approximately normally distributed.

### Equipment

All stimuli were generated digitally in MATLAB (The Mathworks, Natick, MA) with a sampling rate of 48 kHz. The relative level of the five harmonics within a complex tone was controlled before digital-to-analog conversion. The two complex tones (one for each ear) and two TENs (one for each ear) were played out through four channels of a Fireface UCX (RME, Germany) soundcard using 24-bit digital-to-analog conversion, and attenuated independently using four Tucker-Davis Technologies (Alachua, FL) PA4 attenuators. They were then mixed using two Tucker-Davis Technologies SM5 signal mixers, and fed into a Tucker-Davis HB7 headphone driver, which also applied some attenuation, and presented via Sennheiser HD 650 headphones (Wedemark, Germany), which have an approximately diffuse-field response. The specified sound levels are approximate equivalent diffuse-field levels. Listeners were seated individually in a double-walled, sound-insulated booth (IAC, Winchester, UK).

## Data Availability

Correspondence should be addressed to H.E.G. In compliance with our open access requirements, data from this study are available online on request at MRC Cognition and Brain Sciences Unit, https://www.mrc-cbu.cam.ac.uk/publications/opendata/.
